# Ultrasonography-guided reduction of pediatric radial neck fractures

**DOI:** 10.1186/s12891-017-1891-8

**Published:** 2017-12-08

**Authors:** Jung Eun Lee, Jung Bong Kim, Eun Seok Choi

**Affiliations:** Department of Orthopaedic Surgery, Gachon University Gil Hospital, Namdong-daero 774 beon-gil, Namdong-gu, Incheon, 21565 South Korea

**Keywords:** Radial neck fractures, Children, Ultrasonography, Percutaneous pinning

## Abstract

**Background:**

Treatment of displaced and angulated radial neck fractures in children is controversial and challenging. Numerous studies have been conducted regarding treatment algorithms and surgical techniques that use fluoroscopy. However, ultrasonography (US)-guided reduction of pediatric radial neck fractures has not been reported yet. We aimed to determine the safety and efficacy of US-guided reduction and fixation of radial neck fractures in children.

**Methods:**

Among 28 cases of radial neck fracture from 2014 to 2016, 12 were classified as type III or IV according to the Judet classification. All 12 patients underwent US-guided reduction and percutaneous fixation with Kirschner wire and follow-up for more than 6 months. US was used primarily to monitor the angulation and reduction of the radial neck. Fluoroscopy was applied to confirm the fixation with Kirschner wire. Dose area product (DAP; mGy/cm^2^) was measured to assess per-procedure radiation dose. Radiological and clinical results were evaluated at 6 months after the surgery by using the Metaizeau criteria.

**Results:**

Of the patients, 4 were boys and 8 were girls, with a mean age of 7.7 years (range, 5–11 years). Judet type III fractures accounted for 83% of all injuries. The mean preoperative radial angulation was 62.5° (range: 46°–76°). The mean postoperative radial angulation was 5.6° (range: 2°–9°). The mean fluoroscopy time was 31 s (range: 10–73 s), and the mean DAP was 10.7 mGy/cm^2^ (range: 7.2–18.7 mGy/cm^2^). The mean follow-up period was 18.3 months (range, 8–24 months). According to the Metaizeau criteria, 10 cases were excellent and 2 cases were good at the last follow-up.

**Conclusions:**

US-guided reduction and percutaneous fixation is safe and reliable option to treat displaced radial neck fractures in children.

## Background

Radial neck fractures account for approximately 1% of all fractures in children. However, the treatment of displaced and angulated fractures is controversial and challenging [1]. In terms of surgical treatment, several techniques have been documented. Closed surgeries using various reduction techniques, including percutaneous pin or intramedullary elastic nails, have been proposed [1–8]. However, concerns about inadequate reduction, epiphyseal damage, and posterior interosseous nerve injury have emerged [4, 9]. The radial head start to ossify from around age 5 years; if the radial head is mostly cartilaginous, it may be difficult to visualize on radiography. Hence, identification and precise reduction of radial neck fractures by using an intraoperative image intensifier can be limited, and exposure to ionizing radiation can be increased [10].

For the diagnosis of pediatric fractures, the efficacy and reliability of ultrasonography (US) has been demonstrated by several researches [11–14]. However, only few studies have reported the use of US in orthopedic surgeries [15]. We have treated severely displaced radial neck fractures with the percutaneous leverage reduction technique using intraoperative US guidance. To our knowledge, intraoperative use of US for the reduction of radial neck fracture has not been reported yet. The aim of our study was to determine the safety and efficacy of US-guided reduction and fixation of radial neck fractures in children.

## Methods

We retrospectively reviewed the medical records of 28 children with radial neck fractures from 2014 to 2016. Patients with severely angulated radial neck fracture, classified as type III or IV according to the Judet classification, underwent US-guided reduction and fixation with Kirschner wire (K-wire) [5]. The inclusion criteria were as follows: (1) 3–16 years of age, (2) sufficient radiological and medical records, and (3) follow-up for >6 months after surgery. Two patients with multiple fractures and one patient with previous history of supracondylar fracture were excluded. Twelve patients satisfied the inclusion criteria. Medical records, including demographic data, injury characteristics(mechanism of injury, Judet’s classification and direction of displacement), radiation exposures and treatment outcomes were reviewed. Their mean age at surgery was 7.7 years (range, 5–11 years). Of the patients, 4 were boys and 8 were girls. The mean follow-up period was 18.3 months (range, 8–24 months). Slipping down was the most common cause of injury (Table 1). Prior institutional review board (IRB) approval was obtained for this study.

**Table 1:** Clinical characteristics of patients

Number	Age	Side	Mechanism of Injury	Judet Classification	Direction of Displacement	DAP(mGy/cm^2^)	Clinical Result
1	7	Rt	Fall from the height	III	Lateral	15.2	Excellent
2	7	Rt	Slip down	III	Lateral	12.9	Excellent
3	5	Lt	Slip down	III	Lateral	11.6	Excellent
4	5	Rt	Trampoline Injury	III	Lateral	10.5	Excellent
5	6	Rt	Slip down	III	Lateral	8.2	Excellent
6	11	Lt	Slip down	IVa	Lateral	18.7	Good
7	10	Lt	Sports Injury	III	Lateral	7.2	Good
8	5	Lt	Slip down	III	Posterior	8.4	Excellent
9	11	Rt	Slip down	IVa	Posterolateral	12.7	Excellent
10	8	Rt	Fall from the height	III	Lateral	7.2	Excellent
11	8	Lt	Fall from the height	III	Lateral	7.4	Excellent
12	9	Rt	Slip down	III	Lateral	8.2	Excellent

### Surgical techniques

**Fig. 1:**
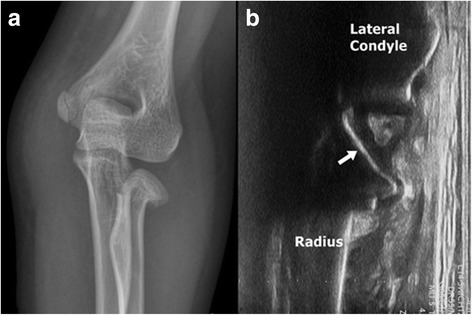
Radiographic and ultrasonographic views of the radial neck fracture. **a** Radial neck fracture Judet grade 3. **b** Intraoperative ultrasonographic view of the fracture. The reverberating echo of the angulated radial head (arrow) is clearly visible

**Fig. 2:**
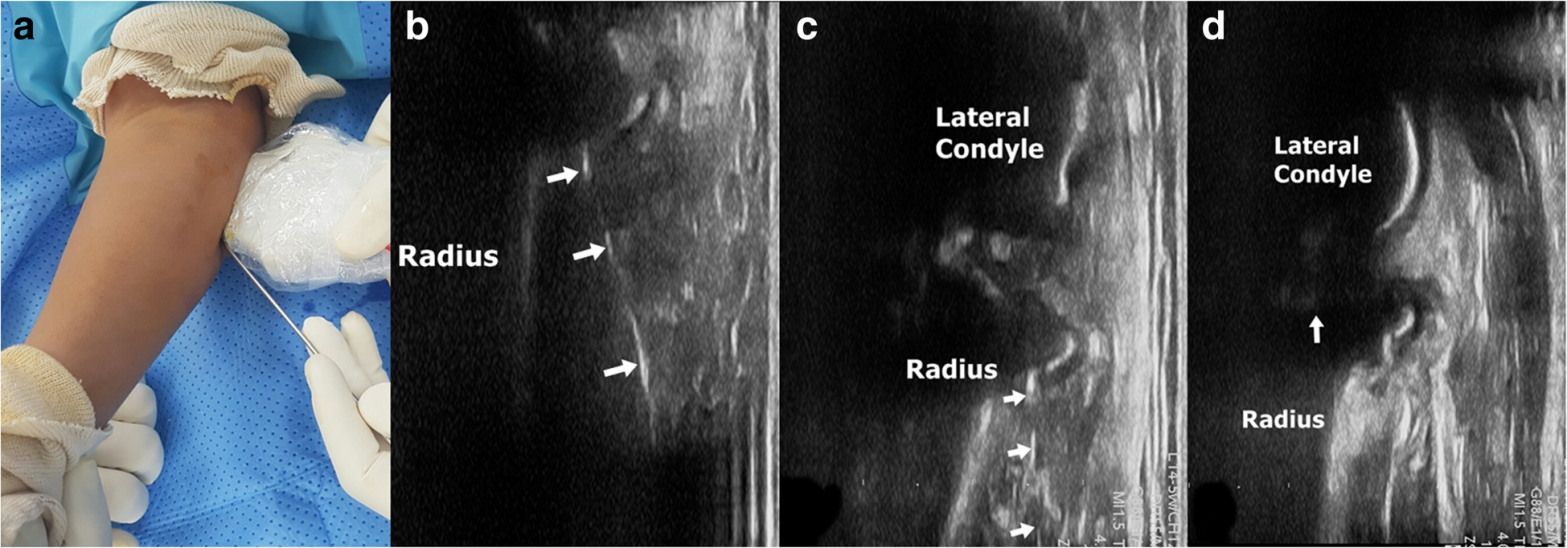
US-guided reduction of radial neck fracture. **a** intraoperative setting of US-guided reduction. **b** Steinman pin(arrows) was introduced from the distal to the radial neck. **c** Reduction of radial neck under the continuous guide of US. **d** The reverberating echo of angulated radial head(Fig. 1b) is disappeared(arrow) after complete reduction

**Fig. 3:**
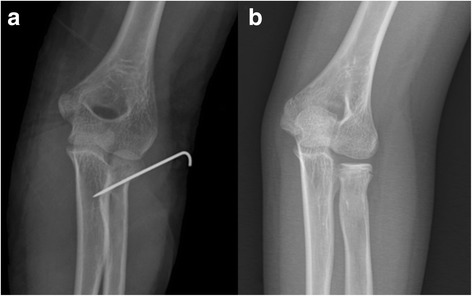
**a** Percutaneous pinning under fluoroscopy guide. **b** Final outcome at 6 months of follow-up

All surgeries were performed at a single institution by one surgeon (ESC). Under general anesthesia, the fractured elbow is extended and the forearm is supinated. A mobile image intensifier (Fluoroscan Insight, Hologic Inc., Bedford, MA/USA) and US (z.one SmartCart, Zonare Medical Systems, Mountain View, CA/USA) were set to be used simultaneously. US was used primarily to monitor the angulation and reduction of the radial neck (Fig. 1). The transducer was placed with its longitudinal axis on the radial shaft. A 2.0-mm Steinmann pin was inserted from the distal and lateral aspects to the fracture site, under US guidance (Fig. 2). US allowed for continuous monitoring of the reduction with a Steinmann pin. After satisfactory reduction was confirmed on US, the radial head is buttressed by the thumb of the operator. We used fluoroscopy for in situ fixation with a second K-wire. The trajectory of the second K-wire was more proximal to the radius than that of the first one and overlapped with the US probe. Moreover, fluoroscopy is essential to check the penetration into the far cortex (Fig. 3). Postoperative care included immobilization using a long arm cast for 3 weeks. The K-wire was removed along with the cast at 3 weeks after surgery in the outpatient clinic. Then, a hinged brace was applied for 3 weeks.

### Postoperative evaluation

Dose area product (DAP; mGy/cm^2^) was measured through the ionization chamber in the mobile image intensifier and used to assess per-procedure radiation dose. Clinical results were evaluated at 6 months after the surgery by using the Metaizeau criteria as follows: excellent, if the fracture healed at the appropriate anatomical position and full range of motion (ROM); good, if the radial neck flexion angle was <20° and the loss of ROM (including flexion, extension, pronation, or supination) was <20° without pain; fair, if the radiological angle was 20–40° and the loss of ROM was <40° with mild instability or pain; poor, if the radiological angle was >40° and the loss of ROM was >40° with residual pain or instability. [5, 16] Statistical analyses were performed using SPSS® software (Version 18.0; SPSS Inc., Chicago, IL, USA).

## Results

Judet type 3 fractures account for 83% of all injuries. The mean preoperative radial angulation was 62.5° (range: 46°–76°). The mean postoperative radial angulation was 5.6° (range: 2°–9°). The mean fluoroscopy time was 31 s (range: 10–73 s), and the mean DAP was 10.7 mGy/cm^2^ (range: 7.2–18.7 mGy/cm^2^).

No nerve injury occurred during the follow-up. Superficial pin site infection occurred in 1 patient but was controlled with oral antibiotics without complications. Long-term complications, including avascular necrosis or synostosis, were not found. According to the Metaizeau reduction classification, 10 cases were excellent and 2 cases were good. One case with good result showed slight angulation (15°) of the radial neck with full ROM at last follow-up (12 months). The other case with good result had limited elbow flexion (10°) without discomfort at last follow-up (12 months).

## Discussion

One of the fundamental ALARA (‘as low as reasonably achievable’) principles is to minimize or avoid radiation whenever possible [17]. Use of US rather than radiography is a representative recommendation based on the ALARA principle, and its advantages for the diagnosis of pediatric fractures has been demonstrated [11–13, 18, 19]. Several reports demonstrated the efficacy and safety of US-guided reduction of fractures at the emergency department. However, only few studies have been conducted regarding the intraoperative use of US for fracture reduction and fixation. As a first report of a US-guided reduction of radial neck fractures, our results demonstrated that US-guided reduction and fixation could be a useful treatment option for pediatric radial neck fractures.

A recent quantification study of radiation exposures in 248 children reported that the mean DAP exposure of supracondylar fractures and flexible nailing of the forearm were 22.3 and 26.7 mGy/cm^2^, respectively [20]. One study documented that the mean DAP of elbow fracture treatments performed by experienced surgeons was 87.41 mGy/cm^2^ [21]. Poor visualization of the radial head and neck, especially in young children, can increase radiation use. In contrast to those studies, we can reduce the radiation exposure (mean DAP: 10.7 mGy/cm^2^) by using US and fluoroscopy in combination.

US has several advantages over radiography in the diagnosis and treatment of pediatric fractures. Fluoroscopy has limitations in identifying pediatric bone with incomplete ossification, especially the radial head of young children [10]. Fractures could be sonographically identified along with subperiosteal hematomas, deformities, cortical disruption, and reverberating echoes [18]. Several researches demonstrated high sensitivity (86.6%–97%) and specificity (86.6%–100%) for fractures of the forearm bone and humerus [12, 13, 19]. In radial neck fractures, the reverberating echo of the radial head is clearly visible and allows for easy assessment of the angulation (Fig. 1). The authors can identify the Steinman pin for reduction and constantly monitor the reduction of the fracture in multiple planes with US (Fig. 2). US can be used to detect associated soft tissue injuries such as tendon injury, hematoma, and nerve injury. However, the use of US for the treatment of fractures has limitations arising from its inability to penetrate the bone. Fluoroscopy is required for bicortical fixation because USG can identify only the near cortex of the bone.

### Limitations

This study has some limitations. The small number of patients included in the study may not be enough to prove the efficacy of our novel technique. Another limitation comes from the design of the study. Although US-guided treatment appears to reduce radiation exposure, further prospective and randomized research is required.

## Conclusion

The present study demonstrated that US-guided reduction can be considered a safe and reliable treatment option for displaced radial neck fractures in children.

## Abbreviations

DAP: Dose area product
US: Ultrasonography
